# Detection Methods of Nanoparticles Synthesized by Gas-Phase Method: A Review

**DOI:** 10.3389/fchem.2022.845363

**Published:** 2022-02-28

**Authors:** Xiushuo Zhang, Xiaolong Zhao, Hongsheng Li, Xiaorui Hao, Jing Xu, Jingjing Tian, Yong Wang

**Affiliations:** ^1^ Laboratory of Optical Detection and Imaging, School of Science, Qingdao University of Technology, Qingdao, China; ^2^ Quantum Physics Laboratory, School of Science, Qingdao University of Technology, Qingdao, China

**Keywords:** gas-phase method, nanoparticles, detection methods, review, expectation

## Abstract

The detection of nanoparticles is the basis of the study of synthesis mechanism, active regulation of the synthesis process, and the study of nanoparticle properties after synthesis. It is significantly meaningful to the academia and engineering industry. Although there are many relevant detection methods at present, each method has its own advantages and disadvantages, and their measurement quantity and application conditions are also different. There is a lack of unified sorting and generalization. In this paper, the significance of detection of nanoparticles synthesized by a gas-phase method is introduced, the development of detection technology is reviewed, and the future is prospected. It is hoped that this paper will provide a reference for the detection of nanoparticles under various conditions and for the development of new detection methods.

## 1 Introduction

Nanoparticles are those particles whose size is between 1 and 100 nm. In a broad sense, nanoparticles are a category of quasi–zero micro–nano materials, which is between a microsystem and macrosystem; they are neither a typical microsystem nor a typical macrosystem. As a result, nanomaterials made from nanoparticles have some amazing properties compared to ordinary materials, which creates conditions for the production of new materials (Yin and Liu, 2006). At present, the gas-phase method is one of the main preparation methods of nanoparticles. It has the advantages of a simple principle, high purity of preparation, large gas contact surface, and thorough reaction. The methods commonly adopted are evaporative condensation (Cui et al., 2010; Chunrui et al., 2017), gas-state reaction (Yu et al., 2005), chemical vapor deposition (Atchudan et al., 2015), sputtering (Jia, 2004), and gas-phase combustion (Dong et al., 2003). And if you want to get high-performance nanomaterials, the particle size, composition, and other information of nanoparticles must be strictly monitored and regulated during the preparation of nanoparticles. Then the properties of nanoparticles were evaluated after preparation. Therefore, the development of corresponding detection technology is of great significance to evaluate the properties of nanomaterials and improve the preparation efficiency and quality of nanoparticles. At present, the detection technology mainly focuses on the measurement of macroscopic statistical characteristics and microstructure characteristics of nanoparticles (Liu et al., 2005). The commonly used detection methods can be divided into two types according to the detection principle: (1) the optical-based detection method; (2) the electrical-based detection method; and (3) the mechanical-based detection method. In this paper, detection methods for nanoparticles are classified into two categories according to timeliness, namely, online detection and off-line detection.

## 2 Off-line Detection of Nanoparticles

### 2.1 The Electrical-Based Detection Method—Electron Microscopy

Electron microscopy is a method which has high visibility and reliability in measuring the size of nanoparticles. Now, electron microscopy is divided into transmission electron microscopy (TEM) and scanning electron microscopy (SEM) (Zheng, 2018). At the same time, it can also be connected with other technologies. Because of these advantages, electron microscopy has become an important method for measuring the size of nanoparticles. Mäkelä (2002) combined TEM and energy-dispersive X-ray spectroscopy (EDX) technology to detect the iodine content collected by carbon films with the probe and achieved good results. The combination of TEM and EDX is the most widely used semi-quantitative analysis technique in off-line detection, and a detection lower limit of 5 nm can be reached. The following sections discuss these two methods separately.

#### 2.1.1 TEM

##### 2.1.1.1 The Principle of TEM

**FIGURE 1:**
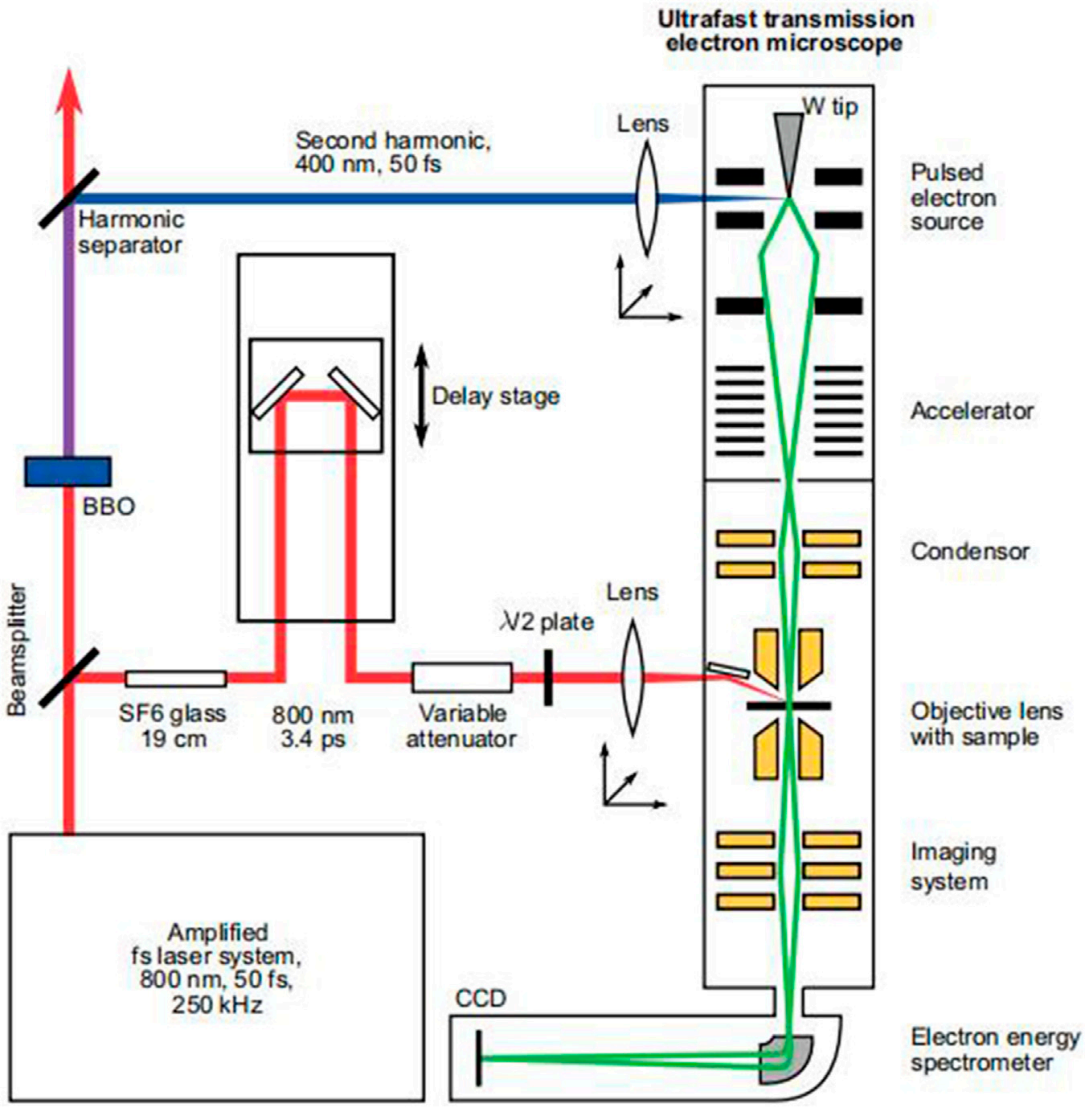
Diagram of the principle of TEM (Feist et al., 2015).

The principle of TEM is based on the interaction between electrons and solids. When electrons pass through the nanoparticle, they undergo elastic and inelastic scattering, carrying information about the object being measured. TEM can collect scattered electrons and extract their information. A Feist et al. introduced the principle of TEM in a paper published in *Nature* in 2015. The images in the A Feist et al. paper are quoted here (Feist et al., 2015), as shown in Figure 1.

##### 2.1.1.2 The Characteristics of TEM

(1) At present, the most advanced TEM has a spatial resolution of less than 0.1 nm and an energy resolution of better than 0.1 eV (Yang et al., 2014).
(2) In addition to the structural information of samples obtained by diffraction and imaging, the chemical composition and electronic structure of samples can be obtained by an energy spectrum (Yang et al., 2014).
(3) TEM is mainly used to observe the surface morphology, dispersion in the matrix, and particle size measurement of nanomaterials.
(4) TEM must operate in a vacuum, as sometimes changing the state of the particle to be measured can affect the measurement.
(5) Compared with other methods, penetrating electron microscopy is a destructive detection.
(6) Due to the small sample size used in electron microscopy observation, its measurement results are often statistically lacking, and the observed particle size distribution range may not represent the whole sample size range (Wang, 2018).

#### 2.1.2 SEM

##### 2.1.2.1 The Principle of SEM

In SEM, a sample is scanned by a focused electron beam that will produce secondary or back-scattered electrons when the focused electron beam hits the sample. These electrons are detected by SEM and converted into images of the sample surface. The particle size of nanoparticles can be obtained by observing the images (James et al., 2010).

**FIGURE 2:**
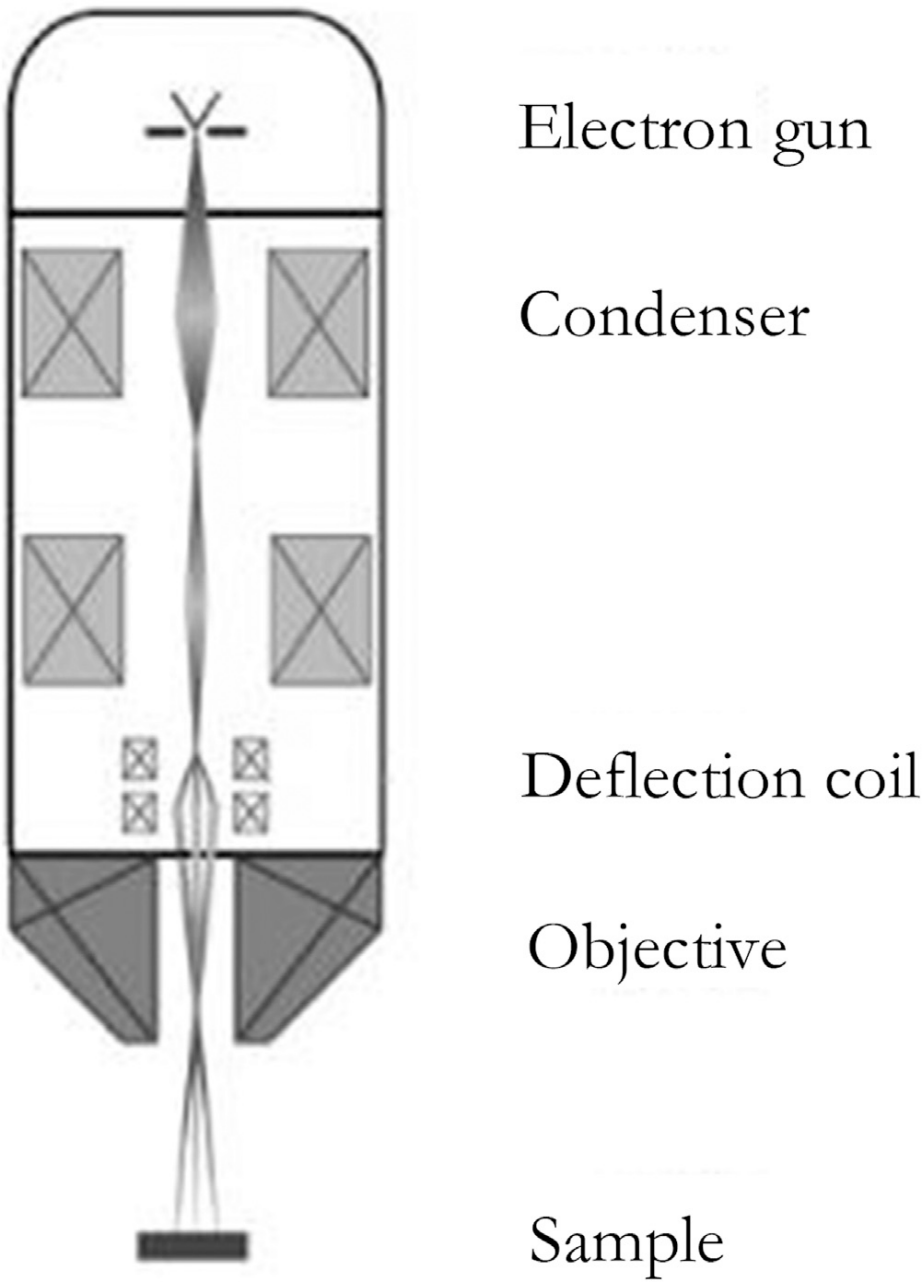
The structure of the SEM (Muzi et al., 2013).

A secondary electron is a kind of free electron produced by the electron beam bombarding the sample so that the outer electrons of the atom in the sample are separated from the atom. The secondary electron has a lower energy, which is generally below 50 eV. As secondary electrons are generated very close to the surface of the sample (generally 5–10 nm away from the surface), secondary electron imaging (SEI) can characterize the sample surface with a high resolution of up to 1 nm (Ling and Zhong, 2018), as shown in Figure 2:

##### 2.1.2.2 The Characteristics of SEM

(1) SEM has an advanced measurement resolution up to 1 nm.
(2) SEM has a wide application range. It not only can measure the particle size of nanoparticles but also can measure the chemical composition of nanoparticles.
(3) SEM has imaging depth of field and good visual field effect, which can provide a clear image for observing the surface topography of the object to be measured.
(4) SEM has a ultra-high resolution that results in a small area or number of nanoparticles being measured at a time, so the measurement results are more accidental and not statistically significant (Yamaguchi et al., 2005).
(5) SEM requires an experimental apparatus that is expensive.

### 2.2 The Mechanical-Based Detection Method

#### 2.2.1 Centrifugal Sedimentation

##### 2.2.1.1 The Principle of Centrifugal Sedimentation

Centrifugal sedimentation is an indirect method to determine particle size distribution. In the static liquid medium, nanoparticles naturally settle by gravity to overcome the resistance and buoyancy of the medium, which causes a change in concentration, pressure, relative density, light transmittance, and settling velocity of the suspension (Chen et al., 2005). These parameters contain information about the particle size and distribution of nanoparticles. According to this, by measuring the variation of these parameters with time, the particle size composition of nanoparticles can be reflected.

##### 2.2.1.2 The Characteristics of Centrifugal Sedimentation

(1) The number of measurements with single measurement is large, so the statistical significance of the measurement results is clear.
(2) With the progress in technology, centrifugal sedimentation was improved to differential centrifugal sedimentation (DCS). The centrifugal sedimentation’s measuring range is within 3 nm–80 μm, and it is easier to use. The analysis speed, accuracy, and repeatability were improved greatly, too.
(3) This method is an indirect measurement method and off-line measurement method.
(4) Centrifugal sedimentation is mostly applied to nanoparticles suspended in liquid, so the liquid-phase method is more widely used.

#### 2.2.2 Atomic Force Microscopy (AFM)

**FIGURE 3:**
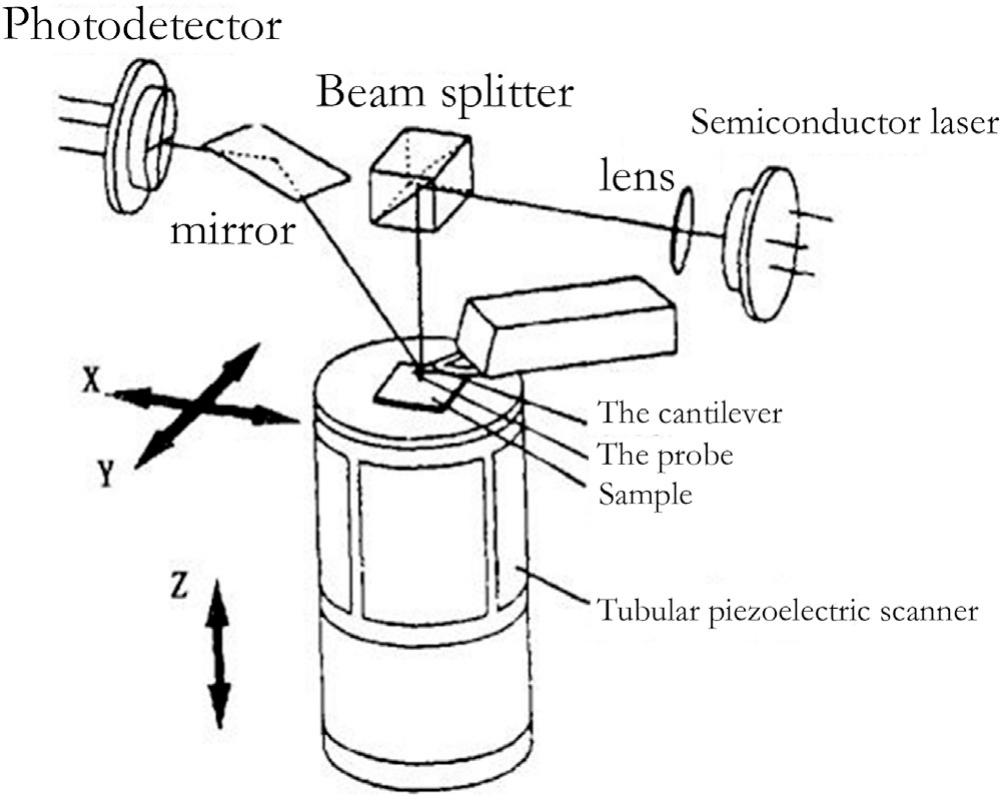
Schematic diagram of AFM (Zhang et al., 2002).

Compared with other existing microscopic tools, AFM has attracted much attention for its features of high resolution, simple sample preparation, and easy operation. It has played an important role in life science, material science, and other fields, greatly promoting the development of nanotechnology and encouraging mankind to enter the nano age. International papers on the research and application of AFM have emerged one after another and have made brilliant achievements (Jie and Sun, 2005). Figure 3 shows the structure of an AFM.

##### 2.2.2.1 The Principle of AFM

The atomic force between atoms is a function of distance, so the surface topography of samples can be obtained by measuring the atomic force of individual atoms. AFM is the measurement of atomic forces on the surface of samples by means of probes. When the atomic force changes, the probe deforms slightly relative to the initial state and is amplified by an optical lever to a position-sensitive detector (PSD). The PSD converts the sample surface topography signal into electrical signal and records the electrical signal. Through continuous measurement, the morphology, particle size, chemical composition, and other characteristics of the samples to be measured are obtained.

##### 2.2.2.2 The Characteristics of AFM (Chunrui et al., 2017)

(1) AFM has a high spatial resolution, and advanced AFM can even reach the sub-angstrom level, which is of great value in the measurement of particle size and chemical composition of nanoparticles.
(2) Compared with traditional electron microscopy, AFM has no requirement for observing materials, while electron microscopy requires that the observed objects must be conductors or coated objects.
(3) Similar to other probe microscopes, AFM is suitable for measuring a single particle, but it is difficult to measure a large number of particles. Meanwhile, due to the small amount of measurement, the statistical significance of the measurement results is insufficient.
(4) AFM can work in vacuum, atmosphere, room temperature, and other different environments; the sample can even be soaked in water and other solutions without special sample preparation technology; and it causes no damage to the sample during the detection process and can carry out contact and non-contact detection.
(5) The sample can be operated by measuring the interaction force between particles and moving atoms.
(6) AFM can be used in combination with other instruments to achieve greater value. AFM-IR technology combines AFM and infrared spectrum for analysis and observation and significantly improves the spatial resolution of AFM through the chemical analysis and composition imaging capability of the infrared spectrum (Dazzi and Prater, 2017).
(7) Currently, AFM also has problems of having a slow imaging speed and low temporal resolution. But at present, research on high-speed AFM is carried out both at home and abroad, and the current advanced AFM can reach more than 10 fps. Yongho Seo et al. (2008) even improved the time resolution of AFM to 30 fps under certain conditions in their paper. It is reasonable to believe that as its frame rate increases, AFM will play an even greater role in future nanoparticle measurement applications.

### 2.3 The Optical-Based Detection Method

#### 2.3.1 X-ray Scattering Techniques

The wavelength of X-ray is similar to the particle size of nanoparticles, so diffraction phenomenon is easy to occur. By analyzing the diffraction image, the information of particle size can be obtained. Currently, there are two main types of X-ray diffraction, X-ray powder diffraction (XRD) and small-angle X-ray scattering. These two methods are described in this paper separately.

##### 2.3.1.1 The Principle of X-ray Scattering Techniques

###### 2.3.1.1.1 XRD

The X-ray diffraction peak is related to the crystal structure of the material, and the crystal structure is directly related to the composition of the material. At the same time, the X-ray diffraction image is also related to the particle size of the nanoparticles. Therefore, the average particle size of nanoparticles can be obtained by studying the X-ray diffraction image (Li et al., 2020).

###### 2.3.1.1.2 Small-Angle X-Ray Scattering

When the X-ray passes through the uneven density distribution of nanoparticles, the X-ray will be scattered at a small angle around the X-ray, and the scattering angle is negatively correlated with the particle size of nanoparticles. Therefore, the size and distribution of nanoparticles can be calculated by measuring the angle of scattering the X-ray.

##### 2.3.1.2 The Characteristics of X-ray Scattering Techniques

###### 2.3.1.2.1 XRD

(1) XRD can measure not only particle size, but also the chemical composition of nanoparticles.
(2) XRD is a non-contact detection method, which does not affect the samples of nanoparticles and belongs to the off-line detection method.
(3) When the particle is a single crystal, the particle size is measured by this method. When grains are polycrystalline, the average grain size of a single grain can be measured by this method. Therefore, the accuracy of the XRD method for particle size measurement is not high, and it cannot identify the aggregation between particles.
(4) XRD is applicable only to the grain size evaluation of crystalline nanoparticles. So the state of the measured nanoparticles has more stringent requirements.

###### 2.3.1.2.2 Small-Angle X-ray Scattering

(1) Small-angle X-ray scattering deals simply with the particles to be measured and is easy to operate.
(2) Small-angle X-ray scattering has a wide measurement range, which can cover the whole particle size range of nanoparticles.
(3) Small-angle X-ray scattering has a high requirement for the morphology of the particles to be measured, and only for single spherical powder, the small-angle X-ray scattering has a high accuracy.
(4) Due to the weak X-ray on the sample to be tested and the distance between the sample and the negative, the exposure time is long and timely detection is not possible. Therefore, small-angle X-ray scattering is considered an off-line detection method.

#### 2.3.2 Raman Spectroscopy

##### 2.3.2.1 The Principle of Raman Spectroscopy

Raman spectroscopy is based on the principle of light scattering. When light hits an object’s surface, different materials produce different Raman scattering or Raman shift. The Raman shift is related to the rotation and vibration of the material, so the Raman shift of the sample to be tested can be measured to identify the size and distribution of the particle size. For the particle size measurement of nanoparticles, it is necessary to measure the peak-to-peak offset between the grain size of nanoparticles and the conventional grain, and the difference can be used to calculate the particle size of nanoparticles.

##### 2.3.2.2 The Characteristics of Raman Spectroscopy

(1) The Raman spectrum is the vibration spectrum of the material itself. Therefore, Raman spectroscopy is specific to the substance, which can measure both particle size and chemical composition of the substance.
(2) Raman spectroscopy is an off-line detection method. This method has high detection sensitivity and is especially suitable for one-dimensional nanomaterial detection. It is easy to operate and does not destroy the sample.

## 3 Online Detection of Nanoparticles

### 3.1 The Electrical-Based Detection Method

#### 3.1.1 Coulter Counter

##### 3.1.1.1 The Principle of Coulter Counter

The Coulter process works by dissolving nanoparticles in an electrolyte and passing them with the electrolyte through a small tube connected to a constant-current circuit. When it passes through the orifice tube, the internal and external resistance of the orifice tube changes instantaneously, resulting in a pulse voltage. The magnitude and number of pulse voltage are positively correlated with the size and number of nanoparticle sample. By measuring the pulse voltage, the particle size and distribution of the sample can be obtained.

##### 3.1.1.2 The Characteristics of Coulter Counter

(1) The Coulter counter is an online detection method. In other words, nanoparticles can be detected during the preparation process, which is of great significance to adjust the preparation process. Compared with other methods, this method has obvious advantages in timeliness and repeatability with higher resolution and shorter measurement time.
(2) The particle size of nanoparticles measured by the Coulter counter is the particle size of the outer surrounding layer of nanoparticles. Therefore, for spherical solid nanoparticles, the measurement accuracy is high, but for spherical shell nanoparticles, the accuracy will be affected.
(3) The Coulter counter is suitable for nanoparticle samples with narrow particle size distribution. For samples with wide particle size distribution, the detection error is large.
(4) The particle size range measured by the Coulter counter is generally above 0.5 μm. Therefore, the measurement ability of the Coulter counter for nanoparticles with a small particle size is insufficient and needs to be improved.
(5) Usually, the measurement environment of the Coulter counter is liquid, so it is difficult to measure gas-phase nanoparticles.

### 3.2 The Mechanical-Based Detection Method

#### 3.2.1 BET (Brunauer, Emmett, and Teller)

##### 3.2.1.1 The Principle of BET

The specific surface area is the ratio of area to mass, reflecting the surface area per unit mass. As you can see by definition and by units, in general, because density is constant, the smaller the mass, the larger the specific surface area. Therefore, the measurement and comparison of the mass-specific surface area can reflect the particle size of nanoparticles. By measuring the mass-specific surface area S_ω_ of nanoparticles, the particle size of nanoparticles can be calculated from Equation 1:

$$D=\frac{6}{\rho S_{\omega }}$$ (1)

In the equation, D is the mass-specific surface area diameter; ρ is the density; and the general measurement method of S_ω_ is the multilayer gas adsorption. And the BET equation is

$$\frac{V}{V_{m}}=\frac{\kappa P}{\left(P_{0}-P\right)\left[1+\left(\kappa -1\right)\frac{P}{P_{0}}\right]}$$ (2)

The entry mode of the sample to be tested by BET is physical adsorption of gas, which could avoid sample contamination caused by chemical adsorption. *V* is the volume of the adsorbed gas; *V*_*m*_ is the volume of gas adsorbed by the monolayer; *P* is the gas pressure; *P*_0_ is the saturated gas pressure; *P*/*P*_0_ is the specific pressure of adsorption; *κ* is *y*/*x*, where *y* = (a_1_/b_1_)*P* and *x* = a_1_/b_1_; and **i** is the adsorption layer. The *V*_*m*_ obtained is converted to the number of adsorbed gas molecules and multiplied by the cross-sectional area A_m_ of an adsorbed gas molecule. The product is the specific surface area S_ω_. Generally speaking, the BET method is used to measure surface area adsorption-specific pressure *P*/*P*_0_ in the range of 0.05–0.35. When the adsorption-specific pressure is less than 0.05, the pressure is too small to establish multilayer physical adsorption equilibrium, or monolayer adsorption cannot even be established. However, when the adsorption-specific pressure is greater than 0.35, the capillary condensation benefit will be generated, and the multilayer physical adsorption equilibrium will be destroyed (Li et al., 2020).

##### 3.2.1 2 The Characteristics of BET

(1) BET has become one of the most widely used and most reliable test results in the industry due to its excellent stability and operability.
(2) The measurement range of specific surface area is about 0.1–1,000 m^2^ g^−1^. For example, the ZrO_2_ powder’s particle size can be measured to 1–10 nm.

### 3.3 The Optical-Based Detection Method

#### 3.3.1 Self-Mixing Interferometry

Foord et al. (1970) first applied PCS (photon correlation spectroscopy) in the study of measuring the diffusion coefficient of hemocyanin at a low concentration. After that, PCS was gradually applied to the detection of nanoparticles and gradually became the mainstream detection method. However, in the detection of high-concentration nanoparticles, due to the complex scattering of particles, the detection error of PCS is large. In order to extend the applicability of PCS, Schtzel (1990) proposed photon cross-correlation spectroscopy (PCCS) to obtain detection data by measuring the fluctuation of the intensity signals of two scattered beams with different angles over time. It could avoid the influence of nanoparticles on the measurement results. And it plays an important role in the detection of nanoparticles. In 2008, Wang and Shen (2008) from the University of Shanghai for Science and Technology and Xuzhou Normal University improved on the basis of and proposed the self-mixing interferometry (SMI). In this method, the light source and detecting element are integrated and packaged to increase the degree of integration. Compared to traditional PCS and PCCS, this design can significantly reduce the error caused by optical path adjustment, improve the signal-to-noise ratio, and enhance the measurement accuracy.

##### 3.3.1.1 The Principle of Self-Mixing Interferometry

**FIGURE 4:**
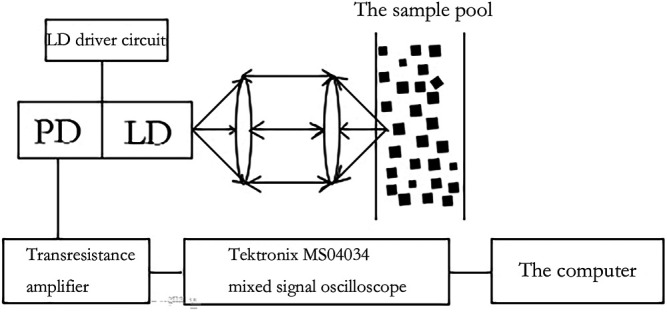
Schematic diagram of laser self-mixing interferometry (Wang and Shen, 2008).

The principle of self-mixing interferometry is that when the laser emits a laser and the incident light hits the Brownian nanoparticles, the light scattered by the particles will have a Doppler shift. This spectrum carries information about the size of the nanoparticles, and some of the light is fed back into the laser resonator. After the self-mixing interferometry of the reflected light and the light in the cavity, the particle size and its distribution can be obtained by measuring the light intensity and power spectrum information. SMI is illustrated in Figure 4 (Wang and Shen, 2008).

##### 3.3.1.2 The Characteristics of Self-Mixing Interferometry

(1) Self-mixing interferometry is greatly convenient for the calibration of the optical path because the light source and detecting element are integrated into one package and simplifies the experimental equipment.
(2) With Fourier transform, the self-mixing interferometry signal is studied in the frequency domain, which avoids the use of a digital correlator and reduces the cost of the experiment and product.
(3) The improved projection algorithm can reverse calculate more accurate particle sizes than before, and the precision is up to 60 nm in this paper.
(4) Self-mixing interferometry has a high signal-to-noise ratio and belongs to the online measurement method.

#### 3.3.2 Laser Particle Size Analysis

Laser particle size analysis mainly uses a laser particle size analyzer, whose principle is light scattering. When the laser shines on the particle, the spatial density and particle size of the nanoparticles play a decisive role in the characteristic parameters of the scattered light. Therefore, the size and distribution of nanoparticles can be calculated by measuring the scattering light intensity and polarization degree. Static light scattering and dynamic light scattering are widely used in laser particle size analysis. However, static light scattering can only measure particle size range above the submicron level. In view of the particle size range of nanoparticles, this section focuses on dynamic light scattering.

##### 3.3.2.1 The Principle of Dynamic Light Scattering

The theoretical basis of the dynamic light scattering method is PCS theory, which enables the laser particle size analysis to detect the particle size range of submicron. However, this leads to the inability to measure the particle size distribution of nanoparticles at the same time. The principle is as follows.

When the particle size of nanoparticles is smaller than the wavelength of the light wave, the angular distribution of the relative intensity of scattered light is no longer affected by the particle size according to the Rayleigh principle. Therefore, static light scattering cannot be used (Sui et al., 2016). The scattered light, on the other hand, has a Doppler shift due to the Brownian motion of the nanoparticles. And because of the smaller particle size of nanoparticles, Brownian motion is more intense, and the effect on the Doppler shift of the scattered light is also stronger (Guo et al., 2006). By measuring the attenuation of the autocorrelation function of the scattering light over time, the particle size information of the nanoparticles can be obtained.

##### 3.3.2.2 The Characteristics of Dynamic Light Scattering

(1) The sample measured by a laser particle size analyzer is not affected by the state; solid, liquid, and gas states can be measured.
(2) The laser particle size analyzer can be used for online detection, which has an important guiding role in the preparation of nanoparticles.
(3) Laser particle size analysis does not destroy the original system of nanoparticles or does not interfere with the original state of samples, which makes it a nondestructive detection method.
(4) Dynamic light scattering can measure the average particle size, which cannot be measured for a single particle. Therefore, in the sample of nanoparticles with wide particle size distribution, the error is large.
(5) Dynamic light scattering is suitable for measuring submicron particle size in the measurement range of 5 nm–2 μm, while static light scattering is suitable for measuring micron particle size.

#### 3.3.3 Improved Method About Dynamic Light Scattering

At present, dynamic light scattering is one of the few methods that can be used for online detection of gas-phase nanoparticles. However, it can only measure the average particle size of a large number of nanoparticles but cannot measure the particle size or chemical composition of a small number or even a single nanoparticle. Therefore, to obtain more detailed nanoparticle information, additional data dimensions must be introduced, and polarized light is the most feasible scheme among them. Polarized light is sensitive to the size, shape, and composition of tiny particles. At the same time, the polarized light path need not be designed separately, as long as the current dynamic light scattering light path-polarizing element can be added. At present, it has been applied to biomedicine (Xiang-Yu et al., 2019; He et al., 2021; Meng et al., 2021; Shen et al., 2021; Song et al., 2021), marine microbial detection (Wang et al., 2018; Li et al., 2020; Liu et al., 2020; Wang et al., 2020; Li et al., 2021), atmospheric detection (Li et al., 2019; Qizhi et al., 2021), etc. It is believed that with the introduction of polarized light technology, the accuracy, measurable parameters, and applicable range of the dynamic light scattering method will be greatly improved.

### 3.4 Other Methods With Different Principles

#### 3.4.1 Ultrasonic Attenuation

##### 3.4.1.1 The Principle of Ultrasonic Attenuation

When the ultrasonic wave propagates in a uniform suspension, it will change with the change of the concentration and particle size of nanoparticles in the tested medium. Ultrasonic transmission is sensitive to the viscosity, temperature, magnetism, and bubbles of the transmission medium, so ultrasonic transmission by the medium will produce scattering loss, viscous inertia dissipation loss, and heat loss, which is called ultrasonic attenuation (Su et al., 2008). The particle size and distribution of nanoparticles can be detected by calculating the ultrasonic attenuation through the theoretical model.

#### 3.4.2 The Characteristics of Ultrasonic Attenuation

(1) Ultrasonic attenuation requires ultrasonic sensors to have full sealing, high permeability, wear resistance, corrosion resistance, and other characteristics.
(2) As an online measurement method, the ultrasonic attenuation spectrum can be measured at the same time as nanoparticles are prepared to ensure the consistency and repeatability of nanoparticles. The current particle size detection range can be up to.
(3) At present, there are many models of ultrasonic attenuation, among which the core–shell model is more suitable for the measurement of the suspension of high-concentration nanoparticles, and the ECAH model is more suitable for the online detection of the particle size of low-concentration nanoparticles.
(4) The ultrasonic attenuation spectrum method needs to dissolve nanoparticles in liquid, so it is suitable for preparing nanoparticles by the liquid method.

#### 3.4.2 Mass Spectrometry

**FIGURE 5:**
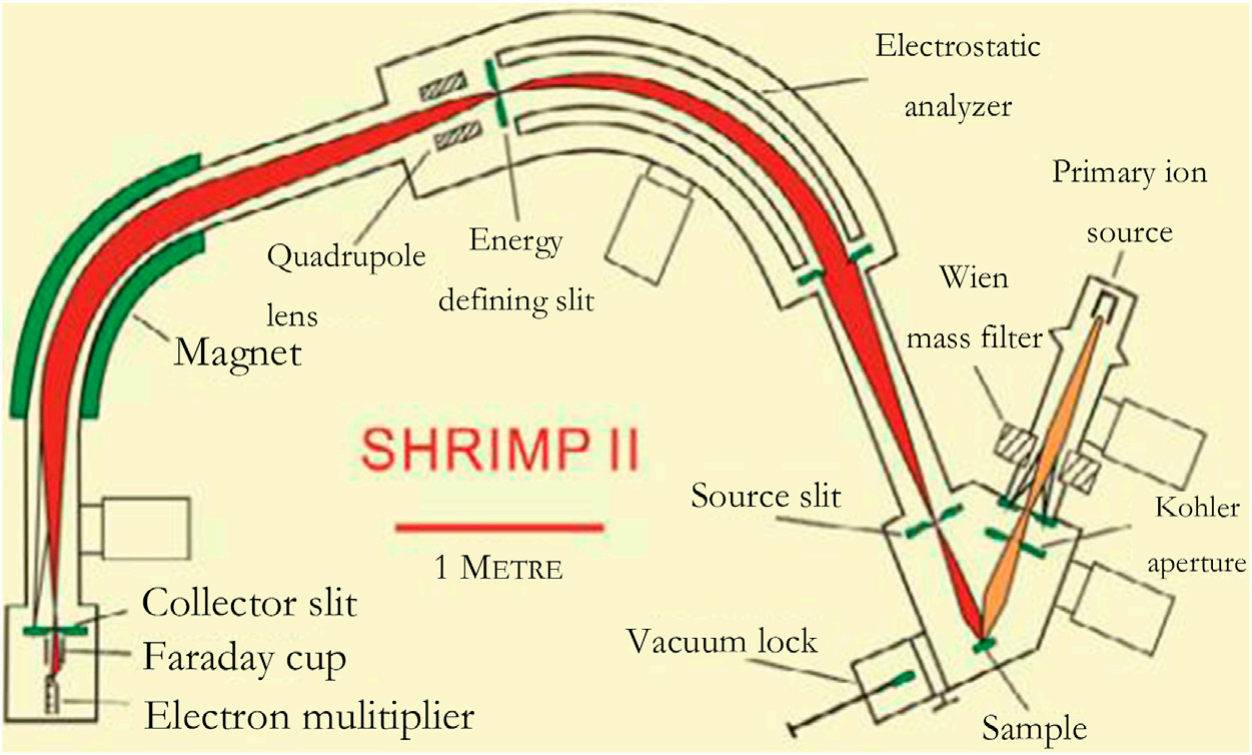
The structure of a secondary ion mass spectrometer (Burgoyne et al., 1997).

The principle of the method using a mass spectrometer as a means of detection is much the same but can be subdivided depending on the feeding method. In this paper, the principle and characteristics of mass spectrometer detection will be introduced, along with several different feeding methods as a separate detection method. Figure 5 shows the structure of a secondary ion mass spectrometer:

##### 3.4.2.1 The Principle of Mass Spectrometry

Mass spectrometers are used to measure the particle size of nanoparticles in gases. According to aerodynamics, nanoparticles are sucked into a mass spectrometer by atmospheric pressure. The nanoparticles with a set value of *m*/*z* were screened out by speed detection devices such as electric field and laser. Then the particles are decomposed into atoms or molecular ions by collision and other methods. After that, these smaller particles will form ion spectra in the mass spectrometer. The particle size and distribution of the nanoparticles can be obtained by analyzing their physical properties such as mass and shape.

##### 3.4.2.2 The Characteristics of Mass Spectrometry

(1) The particle size range determined by mass spectrometry is generally 1–100 nm. It can measure the particle size of nanoparticles with a small particle size, and it also has the upper limit of measurement.
(2) Mass spectrometry is an online detection method. So it can make up for the timeliness information that cannot be obtained by off-line detection methods such as electron microscopy.
(3) In mass spectrometry, the particles need to be decomposed into atoms or ion particles to be measured, so it has a certain destructive effect on the sample.
(4) Different methods are used to measure the detection speed of nanoparticles with different particle sizes. For example, the larger the particle size is, the heavier the mass will be. When the particle size is bigger than 100 nm, the effect of speed detection using electric field and laser will decrease (Motian, 2012).

#### 3.4.3 Aerodynamic Lens

##### 3.4.3.1 The Principle of Aerodynamic Lens

The aerodynamics lens uses a differential air pressure system as a particle sampling device. The differential air pressure system can make the sample to be tested have a smaller particle beam dispersion angle and higher air inlet efficiency, which can significantly improve the instrument’s detection ability. Since the inertia of particles is much greater than that of the surrounding gas molecules, particles tend to converge towards the axis of the lens after multiple lens conversions, while the gas molecules diffuse around during multiple dispersions. For this method, an aerosol mass spectrometer and single particle mass spectrometer are commonly used. When the nanoparticles to be measured into the instrument after the use of a mass spectrometer to achieve the chemical composition of nanoparticle detection.

##### 3.4.3.2 The Characteristics of Aerodynamic Lens

(1) As an online detection method, it can obtain real-time data support and has guiding significance for the preparation of nanoparticles. At the same time, it could serve as a detection method for the preparation of nanoparticles under special conditions.
(2) The method is mainly discussed around the intake process. The traditional aerodynamic lens has a transmission effect for 30–500 nm nanoparticles. However, when the particle size is less than 30 nm, due to the gas expansion and the flow rate not being stable, nanoparticles will also be like air molecules, which cannot gather and diverge around after passing through the lens, and divergence angle and intake efficiency will be greatly affected. Lee et al. (2009) proposed a design scheme of an aerodynamic lens composed of three convergent and divergent holes. Through numerical simulation, his solution can focus the nanoparticles stably under the condition that the airflow is stable and no shock wave is formed. However, in practical application, various conditions are more complex, and the method of Lee et al. should be further improved.
(3) A mass spectrometer is the detection method of an aerodynamics lens. Therefore, it causes certain damage to the samples to be tested and is not suitable for the preparation and detection of nanoparticles for the purpose of use.

#### 3.4.4 Enrichment of Charged Particles

##### 3.4.4.1 The Principle of Enrichment of Charged Particles

According to the principle of electricity, the electric field of specific intensity can screen and enrich charged particles. Therefore, charged nanoparticles can be aggregated in the range below 5 nm, where the aggregation degree of the aerodynamics lens does not work, and they can be detected at the range of 6–20 nm. The method mainly consists of three steps: electrification, enrichment, and detection of nanoparticles. That is, nanoparticles are electrified by an electric charge, and then the particles are concentrated on the wire by means of high voltage. Then the metal wire is moved to the ionization zone, and the enriched particles are thermally volatilized by heating. Finally, these particles will be detected by chemical ionization mass spectrometry.

##### 3.4.4.2 The Characteristics of Enrichment of Charged Particles

(1) The enrichment of charged particles mainly solves the problem of the decrease in the accuracy of the aerodynamics lens in the range below 50 nm and being able to detect at the range of 6–20 nm.
(2) The enrichment of charged particles is mainly used by two kinds of mass spectrometry: one is thermal desorption chemical ionization mass spectroscopy (TD-CIMS) and the other is thermal desorption ion drift chemical ionization mass spectroscopy (TD-ID-CIMS). At present, the main reason that hinders the development of the enrichment of charged particles is that the smaller the particle size is, the more difficult it will be for the charged electric apparatus to charge it, leading to the inability to carry out the next enrichment and measurement. Therefore, Chen et al. (2019) developed a soft X-ray single-stage charger that could replace the charged electric appliance to better charge the nanoparticles to be measured. Meanwhile, Kreisberg et al. (2018) developed a new charged device. By supersaturating the water vapor, the nanoparticles absorb water molecules, increasing their size and thus reducing the difficulty of charging.
(3) The enrichment of charged particles is based on the detection technology of mass spectrometry; it will produce certain damage to the sample. So it is not suitable for chemical composition detection of nanoparticles prepared in small quantities for application purposes.

#### 3.4.5 Electrospray Ionization (ESI)

##### 3.4.5 1 The Principle of ESI

ESI is used to resolve a substance by causing its molecules to form charged ions. The chemical composition of particle was obtained by a mass spectrometer. It is worth mentioning that this method is often used to measure the composition of liquid nanoparticles.

##### 3.4.5.2 The Characteristics of ESI

**FIGURE 6:**
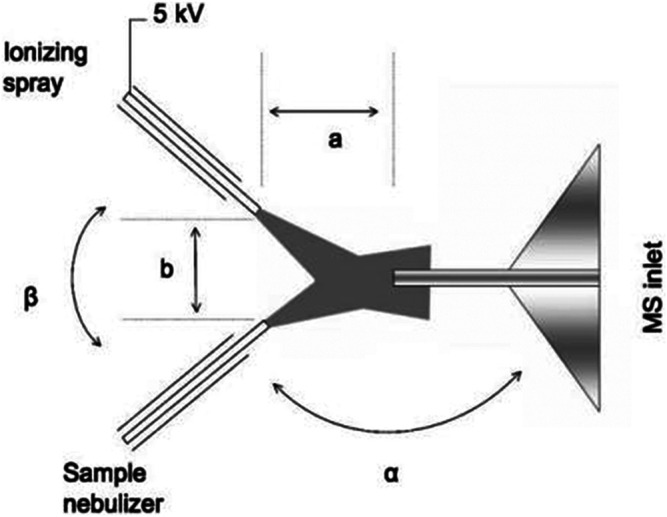
Schematic diagram of extractive ESI (Chen et al., 2006).

(1) Since the ESI method can only detect the ionization of liquid substances, Chen et al. (2006) developed a new electrospray ion source (EESI). It has two separate spray ports, one for the particle spray and the other for the charged droplets, which collide with each other to extract their nanoparticles into the charged droplets. Thus, a new method—electrospray extraction ionization—was developed. It can detect solid, liquid, and gas substances in three states without sample pretreatment. It also can detect the chemical composition of nanoparticles as small as 20 nm. The pictures in their paper are shown in Figure 6.
(2) ESI is also used as the final determination of chemical composition by mass spectrometry. Therefore, it is not suitable for chemical composition detection of nanoparticles prepared in small quantities for application purposes.

## 4 Conclusion

Since Feynman put forward the concept of a nanometer at the end of the 1950s, nanotechnology has been the forefront and hot spot of human scientific research. The emergence of nanomaterials has changed the human understanding of materials, expanded the application scenarios of materials, and become one of the pillars of modern civilization. At present, gas-phase nanoparticles have become an important branch of nanotechnology. However, due to the differences in nanoparticle preparation of the different detection methods, progress in their development has lagged. For example, some methods cannot detect a single particle, some detection methods have insufficient accuracy, and some methods of chemical composition measurement are destructive to samples. These have slowed down the development of vapor-phase nanoparticle technology and even the whole nanotechnology and affected the nanotechnology development prospect. Therefore, it is important to develop the detection technology of gas-phase nanoparticles. At present, the most widely used method for online particle size measurement is the optical method, which uses the scattering or diffraction of light and other principles for measurement. However, the measurement based on the optical principle has advantages in measuring the average particle size of a large number of particles, but it is powerless to measure the particle size of a small number or even a single particle. In the future, more accurate and comprehensive measurement can be achieved by adding measurement dimensions and comprehensively utilizing optical parameters such as intensity, wavelength, phase, and polarized light. At present, the most widely used detection technology for chemical composition is based on mass spectrometry, but the advantages and disadvantages of mass spectrometry are very obvious. The advantages are that the samples can be decomposed, the detection of chemical composition is more accurate, and the chemical composition of gas-phase nanoparticles can be detected, but the disadvantage is also very serious, such as destruction of the sample, which limits it to the detection of only a small amount of nanoparticles prepared for experimental purposes. So it is of great significance to develop online detection for particle size and chemical composition measurement of nanoparticles at present. This will be the basis for the next step in nanotechnology and is worth the attention of researchers.
