# Corrigendum: Detection methods of nanoparticles synthesized by gas-phase method: a review

**DOI:** 10.3389/fchem.2023.1351829

**Published:** 2023-12-14

**Authors:** Xiushuo Zhang, Xiaolong Zhao, Hongsheng Li, Xiaorui Hao, Jing Xu, Jingjing Tian, Yong Wang

**Affiliations:** ^1^ Laboratory of Optical Detection and Imaging, School of Science, Qingdao University of Technology, Qingdao, China; ^2^ Quantum Physics Laboratory, School of Science, Qingdao University of Technology, Qingdao, China

**Keywords:** gas-phase method, nanoparticles, detection methods, review, expectation

In the published article, there was an error in the **Funding** statement. One of the funders was missing. The correct **Funding** statement appears below:

“Open basic research project from the State Key Laboratory of Laser Interaction with Matter, China, No. SKLLIM2021-11; Natural Science Foundation of Shandong Province, China, No. ZR2020QA078; National Natural Science Foundation of China, No. 12005110.”

The authors apologize for this error and state that this does not change the scientific conclusions of the article in any way. The original article has been updated.

